# A Criticism of the Herbst Method of Filling Teeth

**Published:** 1887-01

**Authors:** W. W. Allport

**Affiliations:** Chicago, Ill.


ARTICLE IV.

### A CRITICISM OF THE HERBST METHOD OF FILLING TEETH,

BY W. W. ALLPORT, CHICAGO, ILL.

[Read before the Ohio State Dental Society, Toledo, October, 1886.]

It will be remembered that when Dr. Herbst's method of filling teeth was brought before the American Dental Association a little over two years ago, I then stated, that while not doubting that gold could, under certain conditions be well packed into a cavity in a tooth by it, I was of the opinion that the objections to the system were so many and important that its general adoption in practice was neither practicable nor desirable. The opinions expressed was not received with much favor at the time, nor is it certain that many may agree with me now.

Thinking it possible that there might be good reasons for changing this opinion in regard to the matter, I was glad when it was announced that Dr. Herbst was coming to this country to demonstrate his method, and subsequently, that arrangements had been made for his presence at the late meeting of the American Dental Association that we might see him operate and learn from him directly, his reasons for, and the particular advantages of his method.

The acquaintance I made with Dr. Herbst was certainly a most agreeable one. For him, personally I have the kindest feelings, while for his genius I have a great admiration; and my respect for his head and his heart is such, that I do not believe he would desire you or me to adopt his system of practice, or commend it to others, unless it had the approval of our judgement.

That gold can be well condensed by his system I have not a particle of doubt, nor have I ever had. Neither have I any doubt that most excellent fillings may be made with crystal or plastic gold, for I have put in hundreds and I

may say thousands of them. To be frank in regard to this matter I will say, that much of the little, early reputation I may have gained as an operator, was acquired in the use of crystal gold, for during the first few years of my practice in Chicago I seldom filled a tooth without using more or less of it, this being before the introduction of cohesive foil. But because good operations have been made of it, and can be made of it, is not a sufficient reason for its general use in practice, for experience and observation have taught that quite as good operations can be made with less exactness in details and labor, than is required in the use of this preparation of gold.

With a full acknowledgment of the possibilities that may be attained with this form of gold, the exactness and labor required to reach the highest results in its use are so great, that it was long ago abandoned by a large majority of our best practitioners; though some still make quite extensive use of it and all might possibly make a limited use of it to their advantage. Its general use, however, is neither practicable nor desirable, because quite as good results can be reached with foil, with much less time and labor.

I make this reference to the use of crystal gold for the purpose of illustrating and enforcing my ideas in regard to what is known as the Herbst method.

Without attempting to discuss or explain the physics involved in the impaction of gold by revolving pluggers (burnishers). Accepting the proposition as a demonstrated fact, I will, with your permission, state some of the objections which seem to me, must ever hold against its general adoption in practice.

The first I will mention is the great difficulty and sometimes impossibility of reaching all parts of a cavity with the straight and clumsy instruments, that must be used by this method,—cavities which could be readily reached, and the filling material properly packed with the variously shaped hand or mallet pressure instruments to be found in all well equipped operating cases. In many fissures or deep undercuts that could readily be filled by the old method, a doubt must always be present in the mind of the operator as to whether by this new method such places are perfectly filled or not, for Dr. Herbst himself admits that the only way he can be certain in this matter, is to test the questionable places with small hand-plugger points. It requires no argument to show that a plugger which cannot be relied upon to make known a defective place in a filling, cannot with certainty be relied upon to make the filling good at that point, for an instrument which is too clumsy or ill adapted to find the defect with, is certainly not well adapted to properly fill it with, when found. If hand- or mallet-pluggers can be more safely relied upon to find and fill difficult accessible points about a filling, than can the Herbst method and pluggers, their superiority for filling the balance of the cavity, or for making simple fillings in easily accessible places can hardly be justified.

Second.—To make this method available we are instructed to cut away so much of the sound and strong portions of the tissue as will make all portions of cavities accessible to these arbitrary and clumsy instruments. To this procedure I object and enter an uncompromising protest, for no one has the right to use an instrument or adopt a system of practice that makes it necessary to sacrifice sound, healthy tooth structure, that could be saved by the use of other materials, instruments or methods of practice.

In preparing a cavity to receive a filling, it is the duty of the dentist to remove all tooth structure that cannot be saved in a healthy and useful condition, but no more. He has the right to cut away as much of the sound structure of a tooth as may be necessary to secure a firm foundation and retention of his filling. But in doing this, it is his duty to see to it that the material used for his filling, is, of its kind, the most easily adapted to the intricacies of the cavity to be filled; also that instruments used in packing it are such as are the best calculated to reach all places in the cavity rendered difficult of access, by virtue of saving portions of the tooth, the removal of which would unnecessarily and forever mar its beauty and harmony. To remove more than this to suit his own convenience, or to enable him to use any particular materials or instruments in filling, might, without a very great stretch of imagination or injustice, be considered mal-practice, in a mild form, at least.

While these remarks are made with special reference to the unnecessary sacrifice of tooth substance, by adopting the Herbst method, I will say parenthetically, that they are not without quite too frequent application in regard to fillings put it with cohesive foil. Teeth that could be just as safely filled and much more of the tooth structure saved by a judicious and skillful use of non-cohesive combined with cohesive foil, are in part, mercilessly destroyed in order that the successful use of cohesive foil, exclusively, may be insured.

Admitting, therefore, that under certain circumstances, good fillings may be made by the Herbst method, but to do which, in other cases, would require the destruction of more tooth structure than would be necessary in making good fillings by other well known methods, I believe its general adoption in practice is neither desirable nor justifiable.

Third.—As "time is often said to be the essence of a contract"—an important factor in the consideration—so too, the time consumed in the use of any particular tools, or instruments, in doing a given thing, when contrasted with the time consumed in producing just as good results with other materials and instruments, is an important factor in wisely choosing the instruments or materials to be used.

In making these remarks I am aware that the claim is made by some that much time can be saved by this method; this I do not believe to be true. The same claim was made for crystal gold when it was first introduced and later, for plastic gold when it came up for our approval. Good fillings, we were told, could with old burs, or most any kind of an instrument, be put in with it in from two to three minutes. In neither case, however, has experience proven the claim to have been well founded ; on the contrary more time is consumed in putting in good filling with either of these materials than is required with foil. The same history is to repeat itself in regard to the Herbst method. No doubt fillings can be put in by this process very quickly, but the highest results from it will only be reached by it after much painstaking labor.

If, therefore, as I believe is the fact, under favorable circumstances more time is consumed in making good operations by the Herbst method than by those just mentioned, the method is neither practicable nor desirable in this practical and time-saving age, for time is money, not only to the dentist but also to the patient.

Personally I have had but little experience with these rotary pluggers, but I have seen Dr. Herbst and my friend Dr. Rehwinkel operate, and I have witnessed some of Dr. Marshall's experimental operations by this method, and I confidently assert that I have seen better fillings made by hand mallet, and the old fashioned hand pressure methods, and that too, in much less time than was consumed in making the fillings by the Herbst method. My observation has thus far led me to the belief that there is no saving of time or superiority of operation to be obtained by the new method over the old system. Therefore, as a matter of economy, or superiority of results, I see nothing to justify its general adoption.

Fourth.—That quite as good or even better operations can be made under circumstances favorable for the Herbst method, by the use of suitable instruments and non-cohesive foil enameled with cohesive foil, under mallet pressure, and without the necessity of sacrificing so much sound tooth structure.

While the objections to this method as a general system of filling teeth, are so obvious that its adoption is not warranted, it is possible that as an adjunct to other methods of practice it may be made useful ; in fact I believe it can be. From what I have seen I think it can be made useful at the cervical walls of cavities, especially when the matrix is used. The surface of the pluggers being smooth, and the impact made without violent concussion; when proper access can be had it should be advantageously used in packing gold against margins of cavities, preventing the fracturing of the enamel which so frequently occurs in the use of the mallet pluggers. No doubt it may be advantageously used in other places, but those mentioned are where its greatest excellence will be found. Could a wise judgment be exercised in determining when and where it could, or could not be used with benefit, I have no doubt that it might with advantage be occasionally employed. The fear, however, is that an over zealous advocacy of the system by its friends will lead many to use it when better results could be secured by the old methods. By its general adoption, or too frequent use, frequent failure would be the result, and the method brought into undeserved disrepute, with its abandonment, even while it could be used to advantage, thereby losing, in a measure, at least, the benefits that might follow from a judicious advocacy and use of it.

Now, Mr. President, having stated to you freely and frankly some of my objections to this method of practice, I trust that no one will think I have had the least intention of being unkind or harsh to any one, for nothing could be farther from my desire. I have only intended by words of truth and soberness, to make my views clear to you—to be approved, or disapproved, by argument, or the test of time, as truth may justify.

For Dr. Herbst I have a great admiration. He is more than a Yankee in invention—as generous as King Lear in giving; nor is he so conceited that he cannot learn from others; he freely gives and is wise enough to freely receive. As a profession we are under many obligations to him for the many useful inventions and "tricks," if you please, that he has left with us, and we shall gladly welcome his promised return to this country next year.—*Ohio State Journal.*

